# Fueling Cancer Vaccines to Improve T Cell-Mediated Antitumor Immunity

**DOI:** 10.3389/fonc.2022.878377

**Published:** 2022-05-16

**Authors:** Rosmely Hernandez, Thomas R. Malek

**Affiliations:** Department of Microbiology and Immunology, University of Miami, Miller School of Medicine, Miami, FL, United States

**Keywords:** cancer vaccines, T cells, antitumor immunity, TLR agonists, interleukin-2, checkpoint inhibitors

## Abstract

Cancer vaccines offer the potential to enhance T cell-mediated antitumor immunity by expanding and increasing the function of tumor-specific T cells and shaping the recall response against recurring tumors. While the use of cancer vaccines is not a new immunotherapeutic approach, the cancer vaccine field continues to evolve as new antigen types emerge and vaccine formulations and delivery strategies are developed. As monotherapies, cancer vaccines have not been very efficacious in part due to pre-existing peripheral- and tumor-mediated tolerance mechanisms that limit T cell function. Over the years, various agents including Toll-like receptor agonists, cytokines, and checkpoint inhibitors have been employed as vaccine adjuvants and immune modulators to increase antigen-mediated activation, expansion, memory formation, and T effector cell function. A renewed interest in this approach has emerged as better neoepitope discovery tools are being developed and our understanding of what constitutes an effective cancer vaccine is improved. In the coming years, cancer vaccines will likely be vital to enhance the response to current immunotherapies. In this review, we discuss the various types of therapeutic cancer vaccines, including types of antigens and approaches used to enhance cancer vaccine responses such as TLR agonists, recombinant interleukin-2 and interleukin-2 derivatives, and checkpoint inhibitors.

## 1 Introduction

Almost a century following Edward Jenner’s demonstration in 1796 that protection against smallpox could be achieved through the process of vaccination (1), the American physician William Coley, known as the “Father of Cancer Immunotherapy”, introduced in 1891 the use of vaccines to treat inoperable tumors (2, 3). Using Coley’s method of vaccination, i.e., heat-inactivated Streptococcus pyogenes and Serratia marcescens bacteria, tumor regression and sometimes cures were observed in about 50% of approximately 1000 patients (4, 5). However, this vaccination approach only worked in some cancer types (6). Nevertheless, these initial promising results set the stage for intensive investigation which is still ongoing for more effective cancer vaccines.

Much emphasis initially focused on cancer vaccines that incorporated tumor cells either in the form of lysates or irradiated cells. Modern cancer vaccines typically contain specific tumor antigens related to the patient’s tumor tissue to mount T cell-mediated antitumor immunity. MAGE-A is the first gene reported to encode a tumor antigen, which allowed vaccination against a defined tumor antigen (7). As antigen discovery platforms have improved, many cancer-associated antigens have been molecularly defined. Current vaccines contain these antigens along with other immune modulators to increase antitumor immunity. In this review, we discuss the different types of antigens employed in cancer vaccines and combinatorial strategies used to enhance the response to these vaccines.

## 2 Rationale for Using Tumor Antigen Vaccines to Promote Antitumor T Cell Immunity

Tumor antigens with the potential to activate T cells include non-mutated overexpressed self-proteins, mutated self-proteins, where some contribute to tumorigenicity, and non-self-antigens, e.g., viral proteins from oncogenic viruses. The high overexpression of non-mutated self-antigens sometimes breaks peripheral tolerance, in part due to a high density of self-peptide-MHC complexes that may promote T cell-mediated antitumor responses. Mutated regions of self-antigens and non-self-tumor antigens have a greater likelihood of being more immunogenic in comparison because T cells directed toward such antigens escape thymic negative selection and may express high affinity TCRs. Most current tumor vaccines target these latter types of antigens as these have the potential to elicit stronger antitumor immunity.

Tumor-reactive T cell activity to persistent endogenous tumor antigens can be negatively regulated at the tumor draining lymph nodes and the tumor microenvironment (TME) (8). Despite a high abundance of tumor-derived antigens encountered at these sites, tumor-associated immunosuppressive cues may curtail priming and/or lead to suboptimal cytotoxic responses. Ideal tumor antigen vaccines must avoid immune tolerance mechanisms while inducing tumor-specific immune responses. The antigenic component of these vaccines is administered in the form of peptides and proteins, which may be co-delivered with antigen-presenting cells (APCs), or are encoded in RNA or DNA to prime T cells at more immune permissive sites other than the TME or tumor draining lymph nodes. Additionally, vaccines can be formulated to contain self- or non-self-immunogenic antigens that are highly expressed by the tumor, favoring an effective immune response.

## 3 Cancer Vaccine Antigens

**Figure 1:**
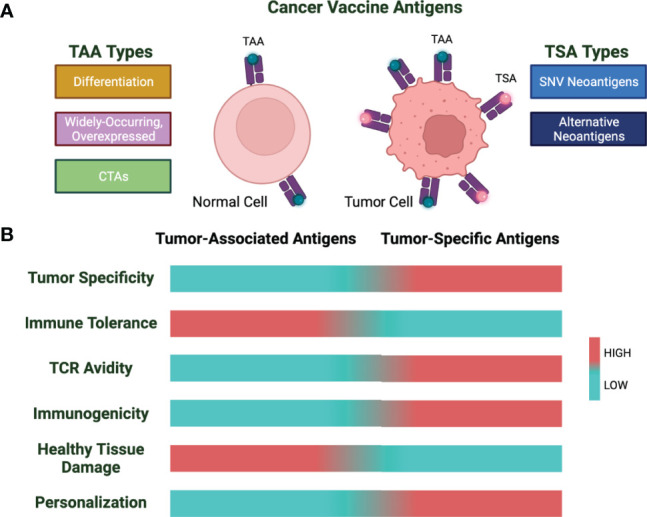
Cancer vaccine antigen types and characteristics. **(A)** Cancer vaccine antigens are divided into tumor-associated antigens (TAA) and tumor-specific antigens (TSA). Shown are subtypes of TAAs and TSAs. **(B)** Characteristics of TAAs and TSAs based on low (cyan) vs. high (red) for the indicated properties.

Tumor vaccines are formulated using two broad categories of cancer antigens: tumor-associated antigens (TAAs) and tumor-specific antigens (TSAs) (**Figure 1**). Of these two, TAAs have been more widely explored as these have been identified more rapidly. However, during the last decade, new technology for the discovery of TSAs and their high immunogenicity has shifted the focus from TAAs to TSAs.

### 3.1 Tumor-Associated Antigens

TAAs are self-antigens that are overexpressed by cancer cells. These are divided into 3 categories: 1) differentiation antigens,2) widely occurring and overexpressed antigens, and 3) cancer testis antigens (CTAs) (9).

Differentiation antigens are proteins that play a tissue-specific function and constitute the majority of TAAs. The most well-known differentiation antigens are associated with melanoma and normal melanocytes, i.e., glycoprotein 100 (gp100), Melan-A/MART-1, and tyrosinase-related proteins. These types of antigens have also been described in breast (mammaglobin-A) and prostate (PSA) cancers (9, 10). Although expressed in normal tissues, vaccines based on differentiation antigens have been widely used for cancer treatment in preclinical and clinical settings. However, clinical findings do not consistently support the use of gp100 and Melan-A/MART-1 TAA vaccines for cancer immunotherapy, as responses are poor and tumor regression is often not achieved. Additionally, in melanoma patients treated with vaccines containing melanocyte-specific antigens, vitiligo, a sign of immune attack to normal melanocytes, has been reported in some studies that often correlates with an anti-tumor response (11–13).

Widely occurring and overexpressed TAAs include tumor suppressor proteins and antiapoptotic proteins (p53, HER-2/neu, livin, and survivin). Although mechanisms of central and peripheral tolerance are very strong for T cells recognizing many TAAs, overexpression or extensive immunization of these TAAs allows those T cells that escape thymic selection to break immune tolerance and become activated by their high antigen density, which in turn leads to tumor attack. The tumor suppressor protein p53 has been widely tested in clinical studies using various vaccine strategies, including p53 peptide-loaded dendritic cells, viral vectors encoding p53, and short and long p53 peptide vaccines (14–17). Although these approaches have generated strong T cell responses, clinical efficacy has also been inconsistent, which in part is related to peripheral immunoregulatory mechanisms that work to restore immune tolerance and inhibit anti-tumor cytotoxic T cell (CTL) activity.

CTAs constitute a safer and more immunogenic vaccine approach. The expression of these TAAs in healthy tissue is restricted to germ cells and placental trophoblasts. Expression of CTAs can be induced in many epithelial tumors, including melanoma, lung, colon, breast, and other carcinomas (9). NY-ESO-1 and MAGE-A represent CTAs with varied expression in these cancer types. The highest frequency of expression of NY-ESO-1 is associated with myxoid and round cell liposarcoma, where nearly 100% of patients with these types of tumors express NY-ESO-1. In contrast, only about 40-50% of patients with melanomas express NY-ESO-1 (18, 19). Although the specific function of CTA re-expression in cancers is not well-understood, high CTA expression in tumors is associated with poor prognosis and advanced disease (20). In some tumors, the highest CTA expression is found in metastases as compared to primary tumors (18, 20, 21). Vaccines targeting the NY-ESO-1 CTA are being used in several clinical studies in combination with adjuvants (19). Additionally, a phase I clinical study tested the safety and feasibility of adoptively transferred CRISPR-Cas9 gene-edited T cells, where the T cell receptor recognizing NY-ESO-1 was introduced while *PD-1* and endogenous *TCR* was knocked out to direct and enhance tumor-recognition in patients with advanced, refractory cancer (22). Although the clinical results suggest safety, additional studies with more patients and improved CRISPR engineering tools must follow to fully assess this therapeutic approach.

Peptide vaccines using TAAs have generated antitumor responses in mouse models. However, clinical responses for this type of peptide-based vaccines have been suboptimal, despite increased immune responses in patients (9, 23, 24). A clear obstacle preventing more robust antitumor responses to vaccines containing TAAs includes the low frequency of T cells specific to the self-antigen and immunoregulatory mechanisms that normally limit self-reactivity. Additionally, vaccinating against TAAs has the potential to generate destructive responses to healthy tissues that also express the TAA.

### 3.2 Tumor-Specific Antigens: Neoantigens

In the past decade, cancer vaccine antigen discovery has moved away from TAAs and toward TSAs. The process of tumor immunoediting has highlighted tumor neoantigens as major inducers of antitumor immunity. Immunoediting is a process by which cancer cells become unrecognizable by the immune system following immune-mediated elimination of tumor cells expressing immunogenic epitopes. The lack of expression of neoantigens by the residual tumor cells importantly contribute to tumor progression (25, 26). For example, in patients with progressive pancreatic ductal adenocarcinoma, highly immunogenic neoantigens expressed by the primary resected tumors were selectively lost in the metastatic lesions of the progressive disease (27). Additionally, in patients with stage IV melanoma treated with adoptive cell therapy or glioblastoma patients receiving PD-1 immune-checkpoint blockade (ICB), neoantigen loss was observed as the tumor progressed, indicating a strong response to neoantigen-expressing tumor cells (28, 29).

TSAs are tumor-specific proteins that arise because of mutations in coding and predicted noncoding regions such as protein-coding gene untranslated regions, pseudogenes, long noncoding RNAs, the antisense strand, and alternative reading frames of the DNA (30). Tumor vaccines comprised of TSAs rather than TAAs provide some potential advantages. Unlike TAAs, neoantigens are thought to be strongly immunogenic as the avidity of TCRs recognizing this class of tumor antigens is usually higher than those TCRs recognizing tumor self-antigens due to the lack of negative selection during central immunological tolerance. Also, targeting tumor neoantigens avoids healthy tissue destruction by T cells as these antigens are only expressed by the tumor tissue (31–36).

One main class of TSAs are non-synonymous single-nucleotide variant (SNV) neoantigens. SNV neoantigens are TSAs that arise from cancer-specific mutations in coding exons and are not present in healthy tissues. Despite the benefits of SNV neoantigens as compared to TAAs, their use as cancer vaccines is personalized and likely preferred for tumors with high mutational burden, which is correlated with neoantigen load. Most SNV neoantigens have been characterized in metastatic melanoma, which is the cancer with highest mutational burden (37). Some studies have also attempted to discover SNV neoantigens in low mutational burden tumors.

Whole exome sequencing technology coupled with RNA sequencing and downstream analyses using mass spectrometry and MHC binding algorithms allows for the identification of SNV neoantigens in mouse and human tumors. Initially, high-throughput sequencing of the B16-F10 mouse melanoma defined SNV neoantigens for vaccine applications (38). Subsequently, SNV neoantigens have been identified in other mouse tumor models, including MC38 and CT26 colon carcinomas, 4T1 breast cancer, TRAMP-C1 prostate cancer, and GL261 and SMA-560 gliomas (39–41). In preclinical studies, vaccination of tumor-bearing mice with SNV neoantigen vaccines showed increased neoantigen-specific T cells that was accompanied by slow disease progression and sometimes tumor rejection in prophylactic and therapeutic settings (38–41). SNV neoantigen vaccination also led to induction of CTL responses against the well-characterized immunodominant AH1 antigen of the CT26 colon carcinoma, indicative of antigen spreading (38, 39). Although neoantigen-prediction approaches have been mainly focused on identifying MHC class I-binding epitopes to induce CTLs, *in silico* selected peptides have been often associated with binding to MHC-class II that generates CD4^+^ T cell responses *in vivo (* 38, 39).

Some clinical studies evaluating the feasibility, safety, and efficacy of personalized neoantigen vaccines have been conducted and show encouraging results. These vaccine platforms have consisted of direct administration of long and short peptides, RNA encoding the neoantigens, or neoantigen-pulsed DC vaccines. Phase I trials have focused mainly on treatment of patients with advanced melanomas (37). The first such trial reported an increase in the frequency of melanoma neoantigen-specific CD8^+^ T cells in PBMCs following administration of a DC vaccine containing melanoma tumor neoantigens (42). Another trial used the NeoVax vaccine, which is comprised of up to 20 neoantigen peptides and adjuvant, to treat patients with resected advanced melanoma. This trial showed neoantigen-specific CD4^+^ T cell-biased responses with transcriptional profiles characteristic of T helper 1 (Th1) and memory T cells (43). Similarly, another Phase I study using an RNA encoding neoantigen vaccine platform showed that the majority of vaccine-elicited responses were mediated by CD4^+^ T cells rather than CD8^+^ T cells (44). More recently, personalized neoantigen vaccines have been studied in patients with glioblastoma, a poorly immunogenic tumor with a low mutational burden (45, 46). Although no clear antitumor efficacy was observed, neoantigen-specific responses following vaccination were reported, demonstrating that neoantigen discovery is also feasible in low-mutational load tumors. Additional trials have been extended to patients with other tumors, including gastrointestinal cancer, non-small cell lung cancer (NSCLC), colorectal cancer, melanoma, pancreatic cancer, biliary tract cancer, ovarian cancer, small-cell lung cancer, adrenal sebaceous adenocarcinoma, breast cancer, parotid carcinoma, and gastric carcinoma (47, 48). These trials also demonstrated that neoantigen vaccines are feasible and safe while eliciting increases in neoantigen-specific T cell responses. However, data regarding antitumor efficacy is limited and more trials must be conducted.

Various other mutational events can give rise to neoantigens (10, 49). These ‘alternative’ TSAs are non-SNV TSAs that include antigens generated by mutational insertion/deletion, frameshifts, endogenous retroviral elements, gene fusions, and post-transcriptionally-derived splice variants (34). Tools to predict alternative TSAs are still being improved and these types of TSA have not been extensively validated in preclinical models. However, as algorithms to predict alternative TSAs become available, these types of antigens might be preferred for vaccine development as some types of alternative TSAs are not patient specific. Additionally, due to the high dissimilarity of alternative TSAs from endogenous antigens, they are expected to exhibit high immunogenicity with a low potential to generate destructive responses to healthy tissue.

Despite the highly immunogenic profile presented by vaccines containing neoantigens, several factors still limit their clinical use and efficacy (50). Although whole exome sequencing technology discovers thousands of nonsynonymous mutations, few of these result in immunogenic neoantigens. Thus, efforts are under way to scan non-coding regions that lead to aberrant peptides to increase neoantigen discovery. Additionally, prediction algorithms must be improved to better identify neoantigens that bind to MHC class I or II molecules. Many neoantigens that were predicted to bind to MHC class I bind MHC class II. Overall, the process of neoantigen development is long and expensive as tumor heterogeneity from patient to patient makes this vaccine approach a highly personalized process.

To evade the problems that come with personalized medicine, some studies are focusing on vaccines that incorporate more “public” neoantigens, i.e., those that are shared by patients with a particular cancer type. These neoantigens are derived from recurrent mutations in tumor driver genes (51). In some gliomas, point mutations in isocitrate dehydrogenase type 1 (*IDH1*) occur during early cancer development. The *IDH1* (R132H) mutation is shared among more than 70% of diffuse grade II and III gliomas and serves as a shared neoantigen. When mice engineered to express human MHC class I and II were vaccinated with this IDH1 neoantigen, a CD4^+^ T cell-dependent immune response limited tumor growth (52). The oncogene *RAS* has been shown to contribute to shared neoantigens. The recurrent Q61K mutation in *RAS* has been defined as a neoantigen shared among 3% of patients with melanoma. The complication that arises with using these public neoantigens is that some of these might not bind to the patients’ HLA alleles (53). In the best scenario, the most potent neoantigen vaccines would incorporate multiple public and “private” or personalized neoantigens to minimize outgrowth of antigen-loss variants.

## 4 Combination Strategies to Improve Cancer Vaccines

**Figure 2:**
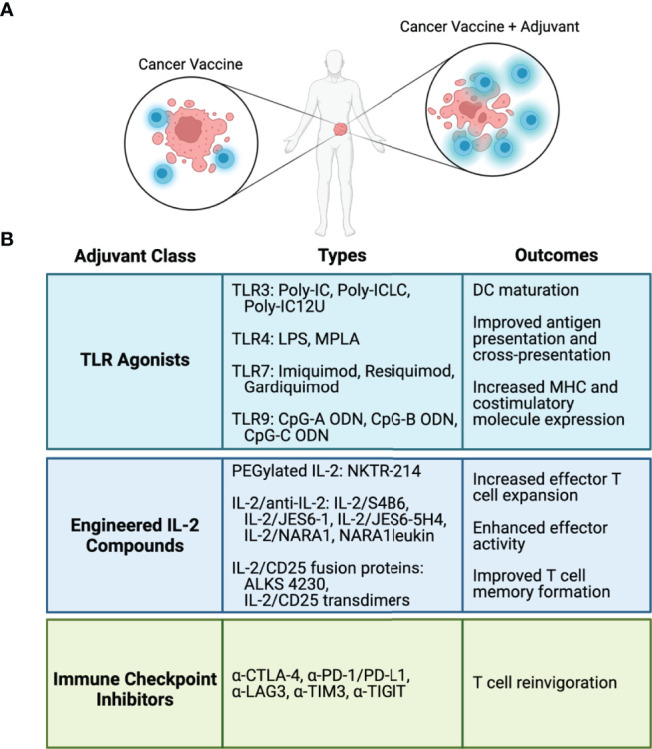
Adjuvants and immune modulators to enhance cancer vaccines. **(A)** Cancer vaccine antigens stimulate antigen-specific T cell-mediated antitumor responses. Adjuvants and immune modulators fortify the T cell response to cancer vaccines. **(B)** Classes of adjuvants and immune modulators, representative types in each class, and biological outcomes from each class.

Despite promising results shown in preclinical and in some clinical studies, the use of cancer vaccines as monotherapies does not lead to robust anti-tumor responses in cancer patients. The lack of effectiveness can be attributed to several factors, including low frequency of endogenous antigen vaccine-specific tumor-reactive T cells, poor antigen presentation and immunogenicity, and immunosuppression by the TME. These findings make plain that these types of vaccines will need to be combined with other immune modulators to enhance the vaccine elicited immune responses. Several ongoing strategies include the use of TLR adjuvants, cytokines, and checkpoint inhibitors (**Figure 2**) and these are considered below.

### 4.1 Toll-Like Receptor Agonists as Cancer Vaccine Adjuvants

TLRs are a class of pattern recognition receptors mostly expressed on the cell surface but can also be found within endosomes and the endoplasmic reticulum (TLR3, 7, 8, and 9) (54). Recognition of agonists by most TLRs on APCs leads to activation of MyD88, resulting in the production of pro-inflammatory cytokines, including TNF, IL-1, and IL-6 (54, 55). Activation of DCs by TLRs induces maturation, stimulation of antigen uptake and processing, and upregulation of MHC and costimulatory molecules, including CD80, CD86, and CD40. Overall, TLR agonists improve antigen presentation and also promote cross-presentation of exogenously derived peptides to stimulate CD8^+^ T cells (55). Thus, antigen vaccines administered to patients in conjunction with TLR adjuvants have increased potential to stimulate CD4^+^ and CD8^+^ T cell-mediated antitumor immunity.

The most well studied TLR agonists in cancer immunotherapy are those which bind to TLR3, 4, 7/8, and 9. Although preclinical studies have shown that several TLR agonists have significant antitumor efficacy, only three TLR agonists have been approved by the FDA. These agonists are BCG for treatment of bladder cancer, Monophosphoryl lipid A (MPLA) for human papillomavirus-induced cervical cancer, and Imiquimod for treatment of basal cell carcinoma (56, 57).

#### 4.1.1 TLR3 Agonists

TLR3 agonists include Poly-IC and derivatives such as Poly-ICLC and Poly-IC12U, where the latter two were developed to improve the safety and immune responses when compared to Poly-IC. These TLR3 agonists are synthetic double-stranded RNA analogs and are used as adjuvant with tumor vaccines. Both Poly-IC and Poly-ICLC signal through TLR3 and melanoma differentiation-associated gene-5 (MDA-5) while Poly-IC12U only signals through TLR3 (58). Signaling induced by Poly-IC and its derivatives leads to DC maturation, production of cytokines (IL-6, IL-12, TNF, IFN-γ), type-I interferons, chemokines (MCP1, MIP1α and MIP1β) and induction of a strong Th1 response (59, 60). When used as an adjuvant to HIV gag peptide, Poly-ICLC was shown to be more potent at inducing a Th1 cellular response than other TLR agonists including LPS, MPLA, CpG, and Resiquimod (58, 61).

Poly-IC and derivatives have been extensively studied in preclinical cancer models and in clinical studies in combination with TAAs. When combined with a prime-boost vaccination regimen with the melanoma TAA Trp1, containing two palmitic acid chains to augment the peptide’s immunogenicity, or with natural Trp2 peptide, Poly-IC enhanced the frequency of mouse antigen-specific CD8^+^ T cells and was more effective than CpG. The resulting immune response supported antitumor immunity to subcutaneously pre-established B16 tumors (62). In combination with costimulatory anti-CD40 antibodies, immunization with Trp2 and Poly-IC led to a strong reduction in metastatic nodules of pre-established B16 melanoma (63). Additionally, Poly-IC used with HPV16-E7 viral peptide induced a CD8^+^ T cell response that led to HPV-induced TC1 tumor rejection in some mice (64). In patients with pancreatic cancer, vaccination with DCs pulsed with the widely, overexpressed self-peptides from telomerase, carcinoembryonic antigen, and survivin together with Poly-ICLC was well tolerated and resulted in increased peptide-specific CD8^+^ T cells (65). The safety and increase in antitumor T cells were also reported in a Phase I trial where patients with ovarian cancer were vaccinated against the NY-ESO-1 antigen in combination with Montanide, an emulsifying agent, and Poly-ICLC. Addition of Poly-ICLC was crucial to obtain the highest antigen-specific immune responses (66).

The benefits of neoantigen vaccines as monotherapies have been limited. Combining MHC class I or II-directed neoantigen vaccines with Poly-IC has significantly improved the neoantigen vaccine antitumor responses to B16-F10 melanoma (38) and MC-38 colon carcinoma (40). Given that Poly-ICLC has demonstrated superiority over other TLR adjuvants in pre-clinical studies with regards to safety and expansion of antigen-specific T cells (38–40, 67), most phase I clinical trials with neoantigen vaccines use Poly-ICLC as the TLR adjuvant to boost the immune response (43, 45, 46, 68). However, as data from neoantigen vaccine trials are still limited, no consensus exists to which TLR adjuvants are best.

#### 4.1.2 TLR4 Agonists

TLR4 is a pattern recognition receptor found on the cell surface that recognizes bacterial lipopolysaccharide (LPS), but also binds to damage associated molecules such as hyaluronan and fibronectin (69). Binding of LPS to TLR4 on APCs induces signaling *via* MyD88 and TRIF, which leads to activation of NF-κB, production of type I interferons and other proinflammatory cytokines, and enhanced antigen processing and presentation on MHC molecules. Multiple studies have shown the antitumorigenic role of TLR4 stimulation. However, some studies suggest that continuous TLR4 simulation, such as that seen in certain inflammatory conditions or chronic infections, can result in tumorigenesis (69).

In humans, bacterial-derived LPS results in toxicities that limit its use. MPLA is a TLR4 agonist derived from LPS that retains the immunostimulatory characteristics of LPS but results in less toxicity (70). MPLA is an FDA approved cancer vaccine adjuvant. Multiples studies have been conducted to determine best route of administration and escalating dosing regimens that promote transient immune activation without the accompanying toxicities.

Vaccines including LPS as an adjuvant lead to increased frequency of effector and memory T cells (71). When LPS was combined with the differentiation TAA gp100 and the interleukin-2 (IL-2) receptor (IL-2R) agonist, IL-2/CD25 transdimers, gp100-specific melanoma-reactive Pmel-1 CD8^+^ T cell expansion, T cell memory formation, and antitumor responses to the B16-F10 mouse melanoma were enhanced to substantially extend mouse survival. When other TLR agonists were used in this setting, Gardiquimod, but not CpG or Poly-IC, supported T cell memory. In the context of tumor vaccines, LPS may improve antitumor immunity in part due to its ability to limit Tregs, which express TLR4 and may dampen the antitumor response (72). LPS is also used as an agent to mature DCs *in vitro* prior to pulsing with peptide antigens to enhance the antigen-presenting capacity of this cellular vaccine (73).

An LPS dose escalation trial was conducted in combination with twelve MHC class I-restricted melanoma peptides, a CD4-activating tetanus helper peptide, and incomplete Freund’s adjuvant (IFA). Combining LPS with these other components was safe at the doses administered and generated a durable T cell response (67). Administration of a vaccine consisting of MPLA and the CTA, MAGE-3, increased the frequency of vaccine-induced CD4^+^ T cells when compared to vaccination with antigen alone in patients with stage I and II non-small cell lung cancer (74).

#### 4.1.3 TLR7 Agonists

TLR7 is expressed in the endosomal compartment and recognizes intracellular pathogens by binding to nucleosides and nucleotides. Due to homology with one of the ligand binding sites of TLR8, the TLR7 agonists, Imiquimod, Resiquimod, and Gardiquimod, also bind TLR8, but to a lesser extent (75, 76). These TLR agonists induce DC maturation, antigen cross-presentation, and production of pro-inflammatory cytokines including IFN-γ, TNF, and IL-2 (77, 78).

Imiquimod is one of the few TLR agonists approved by the FDA as a topical treatment for primary superficial basal cell carcinoma as well as genital warts and actinic keratosis. Despite its value in these conditions, the efficacy of this immune activator is limited when used as a vaccine adjuvant due to its fast diffusion away from the vaccination site (79). However, multiple studies testing this and other TLR7 agonists in the context of cancer vaccines in mice and humans indicate improved vaccine-driven responses.

In a phase IIa clinical study in stage III-IV melanoma patients, Imiquimod in combination with the MelQbG10 vaccine, which contains virus-like nanoparticles loaded with the TLR9 agonist CpG and Melan-A/MART-1 peptide, led to an increase in the frequency of central memory CD8^+^ T cells (80). This increase in memory was enhanced when compared to a previous clinical study using the MelQbG10 vaccine without Imiquimod in stage II-IV melanoma patients (81). No significant toxicities were observed in both studies, indicating that the use of TLR7 agonists in combination with antigen vaccines is safe and triggering both TLR7 and TLR9 in this vaccination modality improves the immune response to the vaccine.

When topical Imiquimod was combined with intradermal administration of the NY-ESO-1 TSA to treat patients with malignant melanoma, NY-ESO-1 CD4^+^, but not CD8^+^, T cell responses were detected (82). The lack of detectable CD8^+^ T cells responsive to NY-ESO-1 was suggested to be due to the timing of Imiquimod administration. However, a later study conducted by the same group to improve upon this trial using Resiquimod showed that only a small number of patients generated CD8^+^ T cells responses to NY-ESO-1 (77). Thus, even though multiple studies have concluded that TLR7 agonists in combination with cancer vaccines are safe and supports antigen-specific responses, optimization of dosing and timing for this class of vaccine adjuvant appears necessary.

More recently, in a preclinical study, the TLR7/8 agonist, Resiquimod (R848), and TLR9 agonist, CpG, were combined in a nanoparticle vaccine containing an MHC class I neoantigen (Adpgk) derived from the mouse MC38 colorectal cancer to elicit antitumor T cell responses (83). This vaccine led to efficient tumor control and improved mouse survival with low toxicity. Tumor control was more pronounced when anti-PD-1 blockade was administered in combination with the nanoparticle vaccine. Thus, combining neoantigen vaccines with TLR agonists and checkpoint inhibitors augments the breadth of the T cell-mediated antitumor response.

#### 4.1.4 TLR9 Agonists

TLR9 is found in late endosomes, lysosomes, and the endoplasmic reticulum. Synthetic oligodeoxynucleotides (ODN) containing unmethylated cytosine-phosphate-guanine (CpG) are TLR9 agonists. Three classes of CpG ODN, Class A, B, and C, have been defined and shown to modulate the immune system differently. Stimulation of TLR9 by CpG-A ODNs induces high amounts of IFN-α and IFN-β by plasmacytoid DCs (pDCs). CpG-B ODNs induce DC maturation and B cell activation but with lower IFN-α and IFN-β production by pDCs. CpG-C ODNs combine the DC and B cell stimulatory activity of Class A and B ODNs (84–86). CpG has been combined with TAAs in preclinical and clinical studies and more recently with TSAs in preclinical mouse tumor models (87–89).

In a phase I trial, the differentiation antigen Melan-A/MART-1 peptide combined with Class B CpG 7909 and IFA was administered to patients with melanoma. This vaccine significantly increased Melan-A/MART-1-specific CD8^+^ T cells when compared to levels prior to treatment and when compared to patients that did not receive CpG as part of the vaccine formulation (90). Addition of CpG to the Melan-A/MART-1 antigen vaccine improved the weak antigen-specific CD8^+^ T cell responses that were observed in previous clinical studies of patients with melanoma receiving Melan-A peptide and IFA (13, 91, 92). Additional phase I clinical studies in patients with stage III-IV melanoma treated with a vaccine containing Melan-A/MART-1 peptide, several other TAAs (gp100 and tyrosinase peptides), CpG, and the emulsifying agent Montanide indicate that CpG supports a higher frequencies of antigen-specific T cells with higher effector function as assessed by increased production of IFN-γ, TNF, and IL-2 (93, 94).

Multiple clinical studies have used CpG to improve NY-ESO-1-driven tumor-specific responses. The consensus has been that this TLR agonist improves the frequency of vaccine-specific T cells, is well tolerated, and in some studies, patient survival is improved (95–98). A recent clinical trial conducted in patients with melanoma showed that vaccination with a long 30-mer peptide from NY-ESO-1, Montanide, and CpG-B is safe and well-tolerated and leads to CD4^+^ and CD8^+^ antigen-specific T cell responses. Despite the strong antigen-reactive immune response, the therapeutic clinical outcome was poor (99).

In preclinical studies, CpG ODNs have also recently been combined with the Adpgk neoantigen from the MC38 colorectal cancer (88). To form adjuvant/antigen nanocomplexes, the neoantigen was modified to contain 10 positively charged lysine residues, which allowed for the self-assembly of nanocomplexes between cationic peptide and anionic CpG. When compared to neoantigen vaccine or CpG alone, these complexes were more effectively taken up by DCs and promoted enhanced DC maturation and cross-presentation. In mice with pre-established MC38 tumors, increased mouse survival was observed as compared to control groups, indicating the potential of this combinatorial approach to improve antitumor immunity. Using the nanoparticle approach, CpG has also improved tumor control of mouse MC38 and CT26 colon carcinomas when combined with mutant KRAS neoantigens, MC38 neoantigens Adpgk and Copg1, and CT26 neoantigens tmem87a and Slc4a3 (89).

### 4.2 Recombinant IL-2 Cytokine to Potentiate Cancer Vaccines

Multiple common gamma chain cytokines, including IL-2, IL-7, IL-15, and IL-21, have been used to enhance cancer vaccine (100). However, of these, IL-2 has been most extensively studied. IL-2 is a cytokine that drives T cell proliferation, differentiation, effector function, survival, and memory formation, but is also essential for Treg development and homeostasis (101). The IL-2R is composed of three subunits: IL-2Rα (CD25), IL-2Rβ (CD122), and the common gamma chain (γc, CD132). Functional IL-2Rs have either intermediate- (CD122/CD132) or high-affinity (CD25/CD122/CD132) for IL-2. The intermediate-affinity IL-2R is mainly expressed by memory-phenotypic CD8^+^ T and NK cells while the high-affinity IL-2R is primarily found on Tregs and recently antigen-activated Teff cells. Vaccination with tumor antigens leads to the formation of the high-affinity IL-2R on activated tumor-reactive T cells (102). Thus, IL-2 has the potential to activate tumor-reactive T effector and memory cells and NK cells to induce antitumor immunity, but also Tregs that may limit inflammation, self-reactivity, and anti-tumor responses. Due to its immunostimulatory activity, high-dose IL-2 was tested in patients with cancer and became the first cytokine approved by the FDA for the treatment of metastatic renal cell carcinoma and metastatic melanoma in 1992 and 1998, respectively (103, 104).

High-dose recombinant IL-2 has been extensively tested in combination with melanoma antigen vaccines. Vaccine regimens incorporating high dose IL-2 as an immune modulator to the melanoma gp100 TAA peptide showed regression of B16 melanoma tumors in mice and increased objective responses in patients with metastatic melanoma as compared to IL-2 or gp100 monotherapy (100, 105–108). Recombinant IL-2 has also been evaluated at a low dose to limit its toxicity to support tumor vaccines. However, when used at a low dose (1 x 10^6^ IU/m^2^/day), IL-2 did not enhance the antitumor response in stage III/IV melanoma patients to a vaccine composed of the NY-ESO-1 CTA, the emulsifier Montanide, and CpG-B (99). Some patients also had adverse events related to this vaccine formulation even though a low IL-2 dose was administered. These adverse events might be attributed, in part, to CpG-B rather than IL-2, as a previous clinical study using higher doses of IL-2 (5 x 10^6^ IU/m^2^/day) combined with Melan-A/MART-1 peptide and the emulsifier, but not CpG, showed lower toxicity and regression of metastases in some patients (92).

#### 4.2.1 IL-2 Analogs to Improve Cancer Vaccines

A major drawback of IL-2 for cancer immunotherapy includes its high toxicity, which often causes life-threatening conditions in patients. High-dosing regimens of IL-2 are required due to its short half-life and because productive Teff responses depend on sustained high IL-2R signaling. However, treatment with high-dose IL-2 leads to off-target effects, particularly Tregs (109), which are considered as a negative prognostic factor in some tumor types by dampening antitumor responses (110, 111). Many new IL-2 analogs, including IL-2 muteins, IL-2-anti-IL-2 antibody complexes, IL-2 modified to contain polyethylene glycol chains, and IL-2-CD25 fusion proteins have been designed with selectivity toward cells expressing the intermediate- or high-affinity IL-2R and improved half-life, which decreases the need for high-dose continuous administrations to limit toxicities. These IL-2 analogs have been described elsewhere (112) and have been tested pre-clinically and some clinically as monotherapies or in combination with cancer vaccines. Their use in the context of cancer vaccines is discussed below.

NKTR-214 is an IL-2 analog containing polyethylene glycol chains that enhance the IL-2 half-life and shift its selectivity toward the intermediate-affinity IL-2R. NKTR-214 has been shown to improve antitumor immunity in mouse cancer models, including the B16-F10 melanoma and the CT26 colon carcinoma when combined with the gp100 self TAA or the endogenous retroviral AH-1 TAA vaccine, respectively (113, 114). In mouse models of B16 melanoma, NKTR-214 improved the expansion and persistence of gp100 vaccinated tumor-specific Pmel-1 T cells. While the frequency of T cell expansion with NKTR-214 was similar to recombinant human IL-2 (~75% vs. ~80%), the magnitude of T cell memory formation was greater for NKTR-214 compared to human IL-2 (~60% vs. ~10%). Although treatment with only the vaccine or NKTR-214 did not support antitumor immunity to B16-F10 melanoma, the combination delayed tumor growth. Similarly, combining NKTR-214 with the AH1 vaccine more effectively limited CT26 growth than the vaccine alone. In a phase 1/2a clinical study (NCT03548467) in patients with locally advanced or metastatic solid tumors, the feasibility, safety, and efficacy of NKTR-214 (Bempegaldesleukin) is being evaluated as an immune modulator to the VB10.NEO DNA plasmid neoantigen vaccine (115).

IL-2/anti-IL-2 complexes, with specificity toward the intermediate- or high-affinity IL-2R, support improved antitumor immunity in comparison to IL-2 when used as a monotherapy (116–118) or as potentiators of tumor vaccines. The IL-2/S4B6 and IL-2/JES6 complexes selective toward the intermediate-affinity or high-affinity IL-2R, respectively, were each more effective than IL-2 in enhancing effector and memory OVA-specific OT-I CD8^+^ induced by OVA-peptide containing vaccines (71, 119). Combining a TriVax vaccine, containing the gp100 TAA, Poly-IC, and anti-CD40 mAb, with another IL-2/anti-IL-2 complex (IL-2/JES6-5H4), with selectivity toward the intermediate-affinity IL-2R, supported enhanced expansion of gp100-specific adoptively transferred Pmel-1 T cells and anti-tumor responses when compared to TriVax monotherapy. In the absence of adoptive transfer of Pmel-1 T cells, TriVax combined with IL-2/JES6-5H4 also led to delayed tumor growth, but to a lesser extent (120). Due to the potent antitumor activity generated by these IL-2/anti-IL-2 complexes in mice, the human IL-2/anti-human IL-2 (hIL-2/NARA1) complex and its derivative, NARA1leukin, were developed for clinical translation. Thus far in pre-clinical studies, IL-2/NARA1 and NARA1leukin complexes induced antitumor immunity as a monotherapy and in combination with mouse melanoma gp100 vaccines (121, 122). When combined with adoptive transfer of *ex-vivo* gp100-stimulated Pmel-1 T cells, IL-2/NARA1 was more effective at expanding these TAA-specific T cells in tumor-draining lymph nodes and tumors compared to PBS- or recombinant human IL-2-treated mice (121). Both IL-2/NARA1 and NARA1leukin halted B16-F10 pulmonary metastasis when combined with a vaccine containing gp100, anti-mouse CD40 antibody, and Poly-IC (122).

At least two IL-2-CD25 fusion proteins, ALKS 4230, targeting the intermediate-affinity IL-2R, and IL-2/CD25 transdimers, targeting the high-affinity IL-2R, have been described with improved antitumor efficacy in mouse B16-F10 melanoma (71, 102, 123). However, data pertaining to the efficacy of these fusion proteins when used as cancer vaccine enhancers is limited to the IL-2/CD25 transdimers fusion protein. When combined with antigen vaccines containing the melanoma TAAs gp100 or trp-1 and LPS or Poly-IC, IL-2/CD25 increased the frequency of effector and memory gp100-specific Pmel-1 CD8^+^ or TRP-1-specific TRP-1 CD4^+^ T cells post adoptive transfer (71, 102). IL-2/CD25 transdimers also improved the frequency of neoantigen-specific T cells following administration of a TSA vaccine containing four B16-F10 melanoma neoantigens and Poly-IC (102). In each setting, the inclusion of IL-2/CD25 improved antitumor immunity when compared to responses after administration of the vaccine alone or the vaccine and recombinant IL-2.

Evidence from studies employing these long-lived IL-2 analogs in combination with cancer vaccines provides a basis for the continued improvement of these combinatorial therapies to further enhance antitumor responses in mouse models and translate these therapies to human studies.

### 4.3 Checkpoint Inhibitors to Support Cancer Vaccines

Under normal physiological conditions or during transient viral infection, inhibitory immune checkpoints regulate the immune response to self-antigens or to viral antigens to promote immune tolerance and limit tissue damage, respectively. In the case of cancer, the expression of inhibitory immune checkpoints limits T cell responses that are crucial to clear the tumor cells. Tumors modulate the T cell response directly through the expression of inhibitory receptors such as PD-L1 or indirectly through production of inhibitory molecules that induce the accumulation of immunosuppressive cells and promote tumor-reactive T cell exhaustion within the TME (124). Several inhibitory immune checkpoints have been described, including CTLA-4, PD-1, PD-L1, LAG3, TIM3, and TIGIT.

Our increased understanding of the mechanisms by which tumors limit T cell responses has led to the consensus that immunotherapeutic approaches must overcome tumor-mediated immune tolerance. One strategy involves using immune checkpoint blockade (ICB). This is an immunotherapeutic approach currently used in the clinic for the treatment of about 50 different cancer types (125). This therapy has significantly improved the treatment of cancers such as melanoma, renal cancer, lung cancer, and others. The most clinically advanced immune checkpoint inhibitors are monoclonal antibodies that antagonize CTLA-4, PD-1 or PD-L1. The overall outcome of ICB is reinvigoration of T cells and enhancement of the antitumor responses (126), which works most effectively for tumors with a high mutation frequency and relatively abundant tumor-neoantigens. For tumors with low mutational burden and a low neoantigen load, ICB might require vaccination with neoantigens to increase the frequency of tumor neoantigen-specific T cells to promote effective antitumor immunity (127, 128). Based on encouraging preclinical data and clinical improvement observed in patients treated with ICB and the immune-stimulating effects of ICB, strategies are under development to combine this ICB with cancer vaccines to improve antigen-specific T cell responses and accumulation of these T cells within the tumor microenvironment.

#### 4.3.1 CTLA-4 Blockade

CTLA-4 blockade has been tested in preclinical cancer mouse models of brain, ovarian, bladder, colon and lung cancer, lymphoma, fibrosarcoma and other cancer types (129). Although in most studies, CTLA-4 blockade monotherapy significantly delayed tumor growth or improved mouse survival, in some cancers including 4T1 breast cancer, MC38 colon cancer, B16 melanoma, lung cancer, and lymphoma, its efficacy has only been demonstrated when combined with cancer vaccines or other therapeutic modalities including monoclonal antibodies, chemotherapeutic drugs, or radiation.

Anti-CTLA-4 has been largely ineffective for the treatment of mouse melanoma as a monotherapy. However, in combination with a trp-2 TAA vaccine and CpG ODN, CTLA-4 ICB increased the survival of mice with pre-established tumors. This combinatorial therapy augmented the numbers of trp-2-specific T cells, where CD4^+^ and CD8^+^ T cells were required for effective antitumor immunity. Neither peptide alone or anti-CTLA-4 and CpG led to antitumor responses that improved mouse survival (130). In a study employing an adenoviral vaccine encoding the gp33-41 epitope of the glycoprotein of lymphocytic choriomeningitis virus (LCMV) to treat gp33-expressing B16-F10 melanoma tumors, blocking CTLA-4 marginally improved tumor control as compared to gp33-adenovirus vaccination alone. The antitumor responses were further improved when anti-CD40 stimulating antibody was added to the vaccination and CTLA-4 ICB (131). Optimal antitumor responses to cellular vaccines consisting of irradiated B16 melanoma cells expressing either GM-CSF or Flt3-ligand (Gvax or Fvax), also depended on ICB (132–134).

CTLA-4 ICB increased the frequencies of gp100- or NY-ESO-1-specific CD8^+^ T cells in melanoma patients after vaccination against the respective antigens. However, the clinical significance of this increase was not clear given the small number of patients tested and variability of the results (135). In another study, gp100 peptide vaccine in IFA did not demonstrate a clinical response as a monotherapy in stage III or IV melanoma patients. ICB with this vaccine did not further increase the overall survival associated with anti-CTLA-4 monotherapy (12). These discouraging results were explained by a later study showing that subcutaneous vaccination with gp100 peptide emulsified in IFA sequestered antigen-primed CD8^+^ T cells at the vaccination site, leading to exhaustion, apoptosis, and reduced number of antigen-specific T cells within the tumor (136, 137). Additional assessment is required to determine whether CTLA-4 ICB in the context of cancer vaccines improves clinical responses. Planned (NCT02950766, NCT03929029, NCT04382664) or ongoing (NCT02275416) clinical trials for the treatment of several cancers, including melanoma and renal cell carcinoma, may provide some guidance.

#### 4.3.2 PD-1/PD-L1 Blockade

PD-1 is an immune checkpoint expressed on some activated T cells, NKT cells, macrophages and monocytes, DCs, and B cells. PD-1 is associated with immune evasion and progression of several cancer types, such as melanoma, renal cell carcinoma, breast cancer, and NSCLC. Its ligands, PD-L1 and PD-L2, are expressed by some tumor and immune cells. Binding of PD-1 to its ligands suppresses T effector responses to limit antitumor immunity (138, 139). PD-1 ICB has been approved by the FDA for treatment of several cancers due to its ability to generate high response rates that improve patient survival (140, 141). Multiple studies indicate that anti-tumor responses by PD-1/PD-L1 ICB are superior to CTLA-4 ICB and result in less toxicity (141, 142).

PD-1/PD-L1 ICB has been tested in preclinical and clinical studies as a vaccine immune modulator to TAAs and TSAs (62, 89, 120, 143, 144). In two independent preclinical studies using vaccines against melanoma-associated TAAs, PD-L1 ICB resulted in improved antitumor efficacy (62, 120). In combination with trp-1 antigen containing two palmitic acid chains and Poly-IC, PD-L1 ICB led to a robust therapeutic effect compared to vaccination alone, which resulted in rejection of subcutaneous melanoma in 80% of the mice (62). Similar to results obtained with the IL-2/JES6-5H4 complex, in the presence or absence of adoptively transferred Pmel-1 T cells, antitumor responses to B16-F10 by TriVax were improved after combination with PD-L1 ICB, suggesting that the vaccine increases the frequency of endogenous gp100-specific T cells and ICB promotes their antitumor activity (120). In neoantigen vaccine studies, PD-1 ICB improved MC38 tumor control elicited by nanoparticle complexes of CpG and neoantigens, including the MC38 neoantigens Adpgk and Copg1 and mutant KRAS neoantigens. This therapy led to a TME dominated by CD8^+^ T cells rather than CD4^+^ T cells (89).

Clinical studies in advanced melanoma patients indicate that blockade of PD-1/PD-L1 improves vaccine responses. In resected advanced melanoma patients, a therapeutic combination of PD-1 ICB and a multi-antigen vaccine containing gp100, MART-1, and NY-ESO-1 peptides was well tolerated, led to increases in peptide-specific T cells, and resulted in low relapse rate (145). For patients that had progressive disease following neoantigen vaccination (43), subsequent PD-1 ICB supported expansion of the neoantigen-specific T cells and complete tumor regression in all patients. This study indicates the value of combining neoantigen vaccines with checkpoint inhibitors. Another approach with promising preclinical findings is combining cancer vaccines and simultaneously blocking CTLA-4 and PD-1. In this regard, a DNA vaccine encoding the OVA or gp100 antigens, IL-12 as an adjuvant, and CTLA-4 and PD-1 combination ICB led to improved antitumor responses compared to single ICB (146).

## 5 Future Perspective

As monotherapies, cancer vaccines, TLR agonists, and engineered IL-2 products will unlikely clear tumors in most patients. Cancer vaccines are crucial to activate and increase the frequency of tumor-reactive T cells, which can be amplified by TLR and IL-2R agonists. ICB has been a major advance for tumor immunotherapy, yet many patients are unresponsive to ICB while others do not achieve long-term durable responses. Thus, application of tumor vaccines in the context of ICB has much potential to significantly improve the outcomes of cancer immunotherapy. An ideal vaccine will lead to high frequency of tumor-reactive CTLs and lead to long-lasting immune memory to protect against tumor recurrence. When combined with ICB, the lowering of immune inhibitory mechanisms will potentiate the vaccine response, limit exhaustion due to persistent tumor antigens, and extend the persistence of highly functional anti-tumor effector cells. Unlike ICB monotherapy, other advantages of this combination approach are that it does not solely rely on pre-existing endogenous tumor-reactive T cells and that less immunogenic tumors with a lower mutation burden may be successfully targeted.

Optimizing cancer vaccines remain paramount to realize this potential. Significant advances have been made to improve antigen discovery platforms for better selection of cancer vaccine antigens, but these need continued refinement, particularly to identify antigens that readily induce tumor-reactive CD8^+^ CTLs. The latest technology has shifted cancer vaccine formulations from those containing self-antigens to neoantigens, as this approach increases immunogenicity while lowering reactivity toward self-tissues. However, vaccines incorporating neoantigens are currently a largely personalized approach, which complicates delivering this approach to large number of patients in a cost-efficient manner.

Effective vaccines must also consider other components beyond the antigen that promote the immune response. Incorporating TLR agonists in the vaccine and delivering IL-2R agonists appears essential. However, current understanding does not clearly point to whether one TLR agonist is preferred or whether the nature or delivery of the neoantigen is critical. Many novel IL-2R agonists have been engineered and some have been shown to amplify the vaccine responses in preclinical studies. Their effectiveness in the clinic remains to be demonstrated. The current view is to use IL-2R agonists that target the intermediate affinity IL-2R expressed by memory-phenotypic CD8^+^ and NK cells to avoid off-target Tregs. In the context of a vaccine, this type of agonist will not show selectivity toward vaccine-induced tumor-reactive CD8^+^ T cells, although some may respond as a result of the bulk expansion of memory-like CD8^+^ T cells. Using IL-2R agonists that target the high-affinity IL-2R may more readily and selectively expand tumor-specific T cells by vaccine-induced upregulation of high-affinity IL-2R, but this comes at the expense of increasing Tregs. For this latter approach to work, the expansion of the tumor-specific T cells must be sufficient to overcome Treg-dependent immunoregulation. As discussed above, recent preclinical studies illustrate the feasibility of this approach to enhance cancer vaccine responses (71, 102). A potential benefit of accompanying Treg expansion after vaccination with a high-affinity IL-2R agonist and ICB is that Tregs might moderate unwanted ICB-dependent autoimmune-like responses.

Although significant progress has been made in the context of cancer vaccine discovery and combinatorial approaches that improve cancer vaccine responses, much work remains to fully realize therapeutic benefits for patients with cancer. Besides defining the best components to elicit effective anti-cancer responses, as discussed above, treatment regimens will also need to be optimized. For example, continued investigation is required to determine not only how frequently to administer the vaccine but also when, how frequently, and at what dose to administer an IL-2R agonist and ICB. An IL-2R agonist might only be needed to generate a critical frequency of tumor-reactive T cells, where subsequent vaccine boosters might be administered independently of the IL-2R agonist. Such an approach could reduce off-target IL-2R responses, including Tregs. Another open question is whether it will be sufficient to switch to ICB monotherapy once a sufficient number of tumor-reactive T cells is achieved or would a treatment regimen with intermittent vaccination and ICB be better. As these issues are resolved, cancer immunotherapy will show ever increasing efficacy and become a first-line option to a greater number of patients.

## Author Contributions

RH wrote the first draft of the article, and TRM edited the article. All authors contributed to the article and approved the submitted version.

## Funding

Our research is supported by grants to TRM from the NIH (NIH R01AI148675, R01AI131648, R21AI159489), the Florida Department of Health (21B03), and a sponsored research agreement with Bristol Myers Squibb. The funder was not involved in the study design, collection, analysis, interpretation of data, the writing of this article or the decision to submit it for publication.

## Conflict of Interest

The University of Miami, TRM, and RH have patents pending on IL-2/CD25 fusion proteins (Wo2016022671A1; TRM) and their use (PCT/US20/13152; TRM, RH) that have been licensed exclusively to Bristol Myers Squibb, and some research on IL-2/CD25 fusion proteins has been supported in part by a collaboration and sponsored research and licensing agreement with Bristol Myers Squibb.

## Publisher’s Note

All claims expressed in this article are solely those of the authors and do not necessarily represent those of their affiliated organizations, or those of the publisher, the editors and the reviewers. Any product that may be evaluated in this article, or claim that may be made by its manufacturer, is not guaranteed or endorsed by the publisher.

## References

[1] Riedel S. Edward Jenner and the History of Smallpox and Vaccination. Proc (Bayl Univ Med Cent) (2005) 18(1):21–5. doi: 10.1080/08998280.2005.11928028 PMC1200696 16200144

[2] Coley WB. The Treatment of Sarcoma With the Mixed Toxins of Erysipelas and Bacillus Prodigiosus. Boston Med Surg J (1908) 158(6):175–82. doi: 10.1056/nejm190802061580601

[3] Coley WB. The Treatment of Malignant Tumors by Repeated Inoculations of Erysipelas. With a Report of Ten Original Cases. 1893. Clin Orthop Relat Res (1991) 262:3–11. doi: 10.1001/jama.1893.02420490019007 1984929

[4] Nauts HC McLaren JR. Coley Toxins–the First Century. Adv Exp Med Biol (1990) 267:483–500. doi: 10.1007/978-1-4684-5766-7_52 2088067

[5] McCarthy EF. The Toxins of William B. Coley and the Treatment of Bone and Soft-Tissue Sarcomas. Iowa Orthop J (2006) 26:154–8. PMC1888599 16789469

[6] Agarwala SS Neuberg D Park Y Kirkwood JM. Mature Results of a Phase III Randomized Trial of Bacillus Calmette-Guerin (BCG) Versus Observation and Bcg Plus Dacarbazine Versus Bcg in the Adjuvant Therapy of American Joint Committee on Cancer Stage I-III Melanoma (E1673): A Trial of the Eastern Oncology Group. Cancer (2004) 100(8):1692–8. doi: 10.1002/cncr.20166 15073858

[7] van der Bruggen P Traversari C Chomez P Lurquin C De Plaen E Van den Eynde B. A Gene Encoding an Antigen Recognized by Cytolytic T Lymphocytes on a Human Melanoma. Science (1991) 254(5038):1643–7. doi: 10.1126/science.1840703 1840703

[8] Thomas SN Vokali E Lund AW Hubbell JA Swartz MA. Targeting the Tumor-Draining Lymph Node With Adjuvanted Nanoparticles Reshapes the Anti-Tumor Immune Response. Biomaterials (2014) 35(2):814–24. doi: 10.1016/j.biomaterials.2013.10.003 24144906

[9] Buonaguro L Petrizzo A Tornesello ML Buonaguro FM. Translating Tumor Antigens Into Cancer Vaccines. Clin Vaccine Immunol (2011) 18(1):23–34. doi: 10.1128/CVI.00286-10 21048000 PMC3019775

[10] Hollingsworth RE Jansen K. Turning the Corner on Therapeutic Cancer Vaccines. NPJ Vaccines (2019) 4:7. doi: 10.1038/s41541-019-0103-y 30774998 PMC6368616

[11] Baba T Sato-Matsushita M Kanamoto A Itoh A Oyaizu N Inoue Y. Phase I Clinical Trial of the Vaccination for the Patients With Metastatic Melanoma Using Gp100-Derived Epitope Peptide Restricted to HLA-A*2402. J Transl Med (2010) 8:84. doi: 10.1186/1479-5876-8-84 20843377 PMC2949666

[12] Hodi FS O'Day SJ McDermott DF Weber RW Sosman JA Haanen JB. Improved Survival With Ipilimumab in Patients With Metastatic Melanoma. N Engl J Med (2010) 363(8):711–23. doi: 10.1056/NEJMoa1003466 PMC3549297 20525992

[13] Cormier JN Salgaller ML Prevette T Barracchini KC Rivoltini L Restifo NP. Enhancement of Cellular Immunity in Melanoma Patients Immunized With a Peptide From Mart-1/Melan a. Cancer J Sci Am (1997) 3(1):37–44. 9072306 PMC2597550

[14] Antonia SJ Mirza N Fricke I Chiappori A Thompson P Williams N. Combination of P53 Cancer Vaccine With Chemotherapy in Patients With Extensive Stage Small Cell Lung Cancer. Clin Cancer Res (2006) 12(3 Pt 1):878–87. doi: 10.1158/1078-0432.CCR-05-2013 16467102

[15] Svane IM Pedersen AE Johansen JS Johnsen HE Nielsen D Kamby C. Vaccination With P53 Peptide-Pulsed Dendritic Cells Is Associated With Disease Stabilization in Patients With P53 Expressing Advanced Breast Cancer; Monitoring of Serum Ykl-40 and Il-6 as Response Biomarkers. Cancer Immunol Immunother (2007) 56(9):1485–99. doi: 10.1007/s00262-007-0293-4 PMC11030002 17285289

[16] Lomas M Liauw W Packham D Williams K Kelleher A Zaunders J. Phase I Clinical Trial of a Human Idiotypic P53 Vaccine in Patients With Advanced Malignancy. Ann Oncol (2004) 15(2):324–9. doi: 10.1093/annonc/mdh053 14760129

[17] Vermeij R Leffers N van der Burg SH Melief CJ Daemen T Nijman HW. Immunological and Clinical Effects of Vaccines Targeting P53-Overexpressing Malignancies. J BioMed Biotechnol (2011) 2011:702146. doi: 10.1155/2011/702146 21541192 PMC3085500

[18] Park TS Groh EM Patel K Kerkar SP Lee CC Rosenberg SA. Expression of Mage-A and Ny-Eso-1 in Primary and Metastatic Cancers. J Immunother (2016) 39(1):1–7. doi: 10.1097/CJI.0000000000000101 26641256 PMC6453128

[19] Thomas R Al-Khadairi G Roelands J Hendrickx W Dermime S Bedognetti D. Ny-Eso-1 Based Immunotherapy of Cancer: Current Perspectives. Front Immunol (2018) 9:947. doi: 10.3389/fimmu.2018.00947 29770138 PMC5941317

[20] Velazquez EF Jungbluth AA Yancovitz M Gnjatic S Adams S O'Neill D. Expression of the Cancer/Testis Antigen NY-ESO-1 in Primary and Metastatic Malignant Melanoma (Mm)–Correlation With Prognostic Factors. Cancer Immun (2007) 7:11. 17625806 PMC2935749

[21] Shantha Kumara HMC Grieco MJ Caballero OL Su T Ahmed A Ritter E. Mage-A3 Is Highly Expressed in a Subset of Colorectal Cancer Patients. Cancer Immun (2012) 12:16. doi: 10.1158/1424-9634.DCL-16.12.2 PMC3554221 23390371

[22] Stadtmauer EA Fraietta JA Davis MM Cohen AD Weber KL Lancaster E. Crispr-Engineered T Cells in Patients With Refractory Cancer. Science (2020) 367(6481):eaba7365. doi: 10.1126/science.aba7365 32029687 PMC11249135

[23] Small EJ Sacks N Nemunaitis J Urba WJ Dula E Centeno AS. Granulocyte Macrophage Colony-Stimulating Factor–Secreting Allogeneic Cellular Immunotherapy for Hormone-Refractory Prostate Cancer. Clin Cancer Res (2007) 13(13):3883–91. doi: 10.1158/1078-0432.CCR-06-2937 17606721

[24] Lipson EJ Sharfman WH Chen S McMiller TL Pritchard TS Salas JT. Safety and Immunologic Correlates of Melanoma Gvax, a GM-CSF Secreting Allogeneic Melanoma Cell Vaccine Administered in the Adjuvant Setting. J Transl Med (2015) 13:214. doi: 10.1186/s12967-015-0572-3 26143264 PMC4491237

[25] O’Donnell JS Teng MWL Smyth MJ. Cancer Immunoediting and Resistance to T Cell-Based Immunotherapy. Nat Rev Clin Oncol (2019) 16(3):151–67. doi: 10.1038/s41571-018-0142-8 30523282

[26] Schreiber RD Old LJ Smyth MJ. Cancer Immunoediting: Integrating Immunity's Roles in Cancer Suppression and Promotion. Science (2011) 331(6024):1565–70. doi: 10.1126/science.1203486 21436444

[27] Balachandran VP Luksza M Zhao JN Makarov V Moral JA Remark R. Identification of Unique Neoantigen Qualities in Long-Term Survivors of Pancreatic Cancer. Nature (2017) 551(7681):512–6. doi: 10.1038/nature24462 PMC6145146 29132146

[28] Verdegaal EM de Miranda NF Visser M Harryvan T van Buuren MM Andersen RS. Neoantigen Landscape Dynamics During Human Melanoma-T Cell Interactions. Nature (2016) 536(7614):91–5. doi: 10.1038/nature18945 27350335

[29] Zhao J Chen AX Gartrell RD Silverman AM Aparicio L Chu T. Author Correction: Immune and Genomic Correlates of Response to Anti-PD-1 Immunotherapy in Glioblastoma. Nat Med (2019) 25(6):1022. doi: 10.1038/s41591-019-0449-8 30996326

[30] Xiang R Ma L Yang M Zheng Z Chen X Jia F. Increased Expression of Peptides From Non-Coding Genes in Cancer Proteomics Datasets Suggests Potential Tumor Neoantigens. Commun Biol (2021) 4(1):496. doi: 10.1038/s42003-021-02007-2 33888849 PMC8062694

[31] Peng M Mo Y Wang Y Wu P Zhang Y Xiong F. Neoantigen Vaccine: An Emerging Tumor Immunotherapy. Mol Cancer (2019) 18(1):128. doi: 10.1186/s12943-019-1055-6 31443694 PMC6708248

[32] Pedersen SR Sorensen MR Buus S Christensen JP Thomsen AR. Comparison of Vaccine-Induced Effector CD8 T Cell Responses Directed Against Self- and Non-Self-Tumor Antigens: Implications for Cancer Immunotherapy. J Immunol (2013) 191(7):3955–67. doi: 10.4049/jimmunol.1300555 24018273

[33] Higgins JP Bernstein MB Hodge JW. Enhancing Immune Responses to Tumor-Associated Antigens. Cancer Biol Ther (2009) 8(15):1440–9. doi: 10.4161/cbt.8.15.9133 PMC7236598 19556848

[34] Smith CC Selitsky SR Chai S Armistead PM Vincent BG Serody JS. Alternative Tumour-Specific Antigens. Nat Rev Cancer (2019) 19(8):465–78. doi: 10.1038/s41568-019-0162-4 PMC6874891 31278396

[35] Hanada KI Yu Z Chappell GR Park AS Restifo NP. An Effective Mouse Model for Adoptive Cancer Immunotherapy Targeting Neoantigens. JCI Insight (2019) 4(10):e124405. doi: 10.1172/jci.insight.124405 PMC6542630 31092734

[36] Gubin MM Artyomov MN Mardis ER Schreiber RD. Tumor Neoantigens: Building a Framework for Personalized Cancer Immunotherapy. J Clin Invest (2015) 125(9):3413–21. doi: 10.1172/JCI80008 PMC4588307 26258412

[37] Alexandrov LB Nik-Zainal S Wedge DC Aparicio SA Behjati S Biankin AV. Signatures of Mutational Processes in Human Cancer. Nature (2013) 500(7463):415–21. doi: 10.1038/nature12477 PMC3776390 23945592

[38] Castle JC Kreiter S Diekmann J Lower M van de Roemer N de Graaf J. Exploiting the Mutanome for Tumor Vaccination. Cancer Res (2012) 72(5):1081–91. doi: 10.1158/0008-5472.CAN-11-3722 22237626

[39] Kreiter S Vormehr M van de Roemer N Diken M Lower M Diekmann J. Mutant Mhc Class Ii Epitopes Drive Therapeutic Immune Responses to Cancer. Nature (2015) 520(7549):692–6. doi: 10.1038/nature14426 PMC4838069 25901682

[40] Yadav M Jhunjhunwala S Phung QT Lupardus P Tanguay J Bumbaca S. Predicting Immunogenic Tumour Mutations by Combining Mass Spectrometry and Exome Sequencing. Nature (2014) 515(7528):572–6. doi: 10.1038/nature14001 25428506

[41] Johanns TM Ward JP Miller CA Wilson C Kobayashi DK Bender D. Endogenous Neoantigen-Specific CD8 T Cells Identified in Two Glioblastoma Models Using a Cancer Immunogenomics Approach. Cancer Immunol Res (2016) 4(12):1007–15. doi: 10.1158/2326-6066.CIR-16-0156 PMC5215735 27799140

[42] Carreno BM Magrini V Becker-Hapak M Kaabinejadian S Hundal J Petti AA. Cancer Immunotherapy. A Dendritic Cell Vaccine Increases the Breadth and Diversity of Melanoma Neoantigen-Specific T Cells. Science (2015) 348(6236):803–8. doi: 10.1126/science.aaa3828 PMC4549796 25837513

[43] Ott PA Hu Z Keskin DB Shukla SA Sun J Bozym DJ. An Immunogenic Personal Neoantigen Vaccine for Patients With Melanoma. Nature (2017) 547(7662):217–21. doi: 10.1038/nature22991 PMC5577644 28678778

[44] Sahin U Derhovanessian E Miller M Kloke BP Simon P Lower M. Personalized Rna Mutanome Vaccines Mobilize Poly-Specific Therapeutic Immunity Against Cancer. Nature (2017) 547(7662):222–6. doi: 10.1038/nature23003 28678784

[45] Hilf N Kuttruff-Coqui S Frenzel K Bukur V Stevanovic S Gouttefangeas C. Actively Personalized Vaccination Trial for Newly Diagnosed Glioblastoma. Nature (2019) 565(7738):240–5. doi: 10.1038/s41586-018-0810-y 30568303

[46] Keskin DB Anandappa AJ Sun J Tirosh I Mathewson ND Li S. Neoantigen Vaccine Generates Intratumoral T Cell Responses in Phase Ib Glioblastoma Trial. Nature (2019) 565(7738):234–9. doi: 10.1038/s41586-018-0792-9 PMC6546179 30568305

[47] Fang Y Mo F Shou J Wang H Luo K Zhang S. A Pan-Cancer Clinical Study of Personalized Neoantigen Vaccine Monotherapy in Treating Patients With Various Types of Advanced Solid Tumors. Clin Cancer Res (2020) 26(17):4511–20. doi: 10.1158/1078-0432.CCR-19-2881 32439700

[48] Cafri G Gartner JJ Zaks T Hopson K Levin N Paria BC. Mrna Vaccine-Induced Neoantigen-Specific T Cell Immunity in Patients With Gastrointestinal Cancer. J Clin Invest (2020) 130(11):5976–88. doi: 10.1172/JCI134915 PMC7598064 33016924

[49] Blass E Ott PA. Advances in the Development of Personalized Neoantigen-Based Therapeutic Cancer Vaccines. Nat Rev Clin Oncol (2021) 18:215–29. doi: 10.1038/s41571-020-00460-2 PMC7816749 33473220

[50] Zhang Z Lu M Qin Y Gao W Tao L Su W. Neoantigen: A New Breakthrough in Tumor Immunotherapy. Front Immunol (2021) 12:672356. doi: 10.3389/fimmu.2021.672356 33936118 PMC8085349

[51] Pearlman AH Hwang MS Konig MF Hsiue EH-C Douglass J DiNapoli SR. Targeting Public Neoantigens for Cancer Immunotherapy. Nat Cancer (2021) 2(5):487–97. doi: 10.1038/s43018-021-00210-y PMC8525885 34676374

[52] Schumacher T Bunse L Pusch S Sahm F Wiestler B Quandt J. A Vaccine Targeting Mutant Idh1 Induces Antitumour Immunity. Nature (2014) 512(7514):324–7. doi: 10.1038/nature13387 25043048

[53] Peri A Greenstein E Alon M Pai JA Dingjan T Reich-Zeliger S. Combined Presentation and Immunogenicity Analysis Reveals a Recurrent Ras.Q61k Neoantigen in Melanoma. J Clin Investig (2021) 131(20):e129466. doi: 10.1172/JCI129466 34651586 PMC8516471

[54] Kawai T Akira S. Tlr Signaling. Semin Immunol (2007) 19(1):24–32. doi: 10.1016/j.smim.2006.12.004 17275323

[55] Adams S. Toll-Like Receptor Agonists in Cancer Therapy. Immunotherapy (2009) 1(6):949–64. doi: 10.2217/imt.09.70 PMC2886992 20563267

[56] Urban-Wojciuk Z Khan MM Oyler BL Fåhraeus R Marek-Trzonkowska N Nita-Lazar A. The Role of Tlrs in Anti-Cancer Immunity and Tumor Rejection. Front Immunol (2019) 10:2388(2388). doi: 10.3389/fimmu.2019.02388 31695691 PMC6817561

[57] Vacchelli E Galluzzi L Eggermont A Fridman WH Galon J Sautès-Fridman C. Trial Watch: Fda-Approved Toll-Like Receptor Agonists for Cancer Therapy. Oncoimmunology (2012) 1(6):894–907. doi: 10.4161/onci.20931 23162757 PMC3489745

[58] Trumpfheller C Caskey M Nchinda G Longhi MP Mizenina O Huang Y. The Microbial Mimic Poly Ic Induces Durable and Protective CD4^+^ T Cell Immunity Together With a Dendritic Cell Targeted Vaccine. Proc Natl Acad Sci U.S.A. (2008) 105(7):2574–9. doi: 10.1073/pnas.0711976105 PMC2268178 18256187

[59] Jasani B Navabi H Adams M. Ampligen: A Potential Toll-Like 3 Receptor Adjuvant for Immunotherapy of Cancer. Vaccine (2009) 27(25-26):3401–4. doi: 10.1016/j.vaccine.2009.01.071 19200817

[60] Martins KAO Bavari S Salazar AM. Vaccine Adjuvant Uses of Poly-IC and Derivatives. Expert Rev Vaccines (2015) 14(3):447–59. doi: 10.1586/14760584.2015.966085 25308798

[61] Longhi MP Trumpfheller C Idoyaga J Caskey M Matos I Kluger C. Dendritic Cells Require a Systemic Type I Interferon Response to Mature and Induce CD4^+^ Th1 Immunity With Poly Ic as Adjuvant. J Exp Med (2009) 206(7):1589–602. doi: 10.1084/jem.20090247 PMC2715098 19564349

[62] Cho H-I Barrios K Lee Y-R Linowski AK Celis E. Bivax: A Peptide/Poly-Ic Subunit Vaccine That Mimics an Acute Infection Elicits Vast and Effective Anti-Tumor CD8 T-Cell Responses. Cancer Immunol Immunother (2013) 62(4):787–99. doi: 10.1007/s00262-012-1382-6 PMC3625508 23266830

[63] Cho HI Celis E. Optimized Peptide Vaccines Eliciting Extensive CD8 T-Cell Responses With Therapeutic Antitumor Effects. Cancer Res (2009) 69(23):9012–9. doi: 10.1158/0008-5472.Can-09-2019 PMC2789207 19903852

[64] Barrios K Celis E. Trivax-Hpv: An Improved Peptide-Based Therapeutic Vaccination Strategy Against Human Papillomavirus-Induced Cancers. Cancer Immunol Immunother (2012) 61(8):1307–17. doi: 10.1007/s00262-012-1259-8 PMC3446251 22527249

[65] Mehrotra S Britten CD Chin S Garrett-Mayer E Cloud CA Li M. Vaccination With Poly(Ic:Lc) and Peptide-Pulsed Autologous Dendritic Cells in Patients With Pancreatic Cancer. J Hematol Oncol (2017) 10(1):82. doi: 10.1186/s13045-017-0459-2 28388966 PMC5384142

[66] Sabbatini P Tsuji T Ferran L Ritter E Sedrak C Tuballes K. Phase I Trial of Overlapping Long Peptides From a Tumor Self-Antigen and Poly-Iclc Shows Rapid Induction of Integrated Immune Response in Ovarian Cancer Patients. Clin Cancer Res (2012) 18(23):6497–508. doi: 10.1158/1078-0432.Ccr-12-2189 23032745

[67] Melssen MM Petroni GR Chianese-Bullock KA Wages NA Grosh WW Varhegyi N. A Multipeptide Vaccine Plus Toll-Like Receptor Agonists Lps or Polyiclc in Combination With Incomplete Freund’s Adjuvant in Melanoma Patients. J Immunother Cancer (2019) 7(1):163. doi: 10.1186/s40425-019-0625-x 31248461 PMC6598303

[68] Chen X Yang J Wang L Liu B. Personalized Neoantigen Vaccination With Synthetic Long Peptides: Recent Advances and Future Perspectives. Theranostics (2020) 10(13):6011–23. doi: 10.7150/thno.38742 PMC7255011 32483434

[69] Awasthi S. Toll-Like Receptor-4 Modulation for Cancer Immunotherapy. Front Immunol (2014) 5:328. doi: 10.3389/fimmu.2014.00328 25120541 PMC4110442

[70] Albert Vega C Karakike E Bartolo F Mouton W Cerrato E Brengel-Pesce K. Differential Response Induced by LPA and MPLA in Immunocompetent and Septic Individuals. Clin Immunol (2021) 226:108714. doi: 10.1016/j.clim.2021.108714 33741504

[71] Hernandez R Toomer KH Poder J Santos Savio A Hsiung S Malek TR. Sustained IL-2r Signaling of Limited Duration by High-Dose mIL-2/Mcd25 Fusion Protein Amplifies Tumor-Reactive CD8^+^ T Cells to Enhance Antitumor Immunity. Cancer Immunol Immunother (2021) 70(4):909–21. doi: 10.1007/s00262-020-02722-5 PMC7979461 33037893

[72] Hsiung S Moro A Ban Y Chen X Savio AS Hernandez R. Acute Lipopolysaccharide-Induced Inflammation Lowers IL-2r Signaling and the Proliferative Potential of Regulatory T Cells. Immunohorizons (2020) 4(12):809–24. doi: 10.4049/immunohorizons.2000099 33334814

[73] Yazdani M Gholizadeh Z Nikpoor AR Mohamadian Roshan N Jaafari MR Badiee A. *Ex Vivo* Dendritic Cell-Based (DC) Vaccine Pulsed With a Low Dose of Liposomal Antigen and CpG-ODN Improved PD-1 Blockade Immunotherapy. Sci Rep (2021) 11(1):14661. doi: 10.1038/s41598-021-94250-0 34282215 PMC8290007

[74] Atanackovic D Altorki NK Stockert E Williamson B Jungbluth AA Ritter E. Vaccine-Induced CD4^+^ T Cell Responses to MAGE-3 Protein in Lung Cancer Patients. J Immunol (2004) 172(5):3289–96. doi: 10.4049/jimmunol.172.5.3289 14978137

[75] Ma F Zhang J Zhang J Zhang C. The TLR7 Agonists Imiquimod and Gardiquimod Improve DC-Based Immunotherapy for Melanoma in Mice. Cell Mol Immunol (2010) 7(5):381–8. doi: 10.1038/cmi.2010.30 PMC4002679 20543857

[76] Chi H Li C Zhao FS Zhang L Ng TB Jin G. Anti-Tumor Activity of Toll-Like Receptor 7 Agonists. Front Pharmacol (2017) 8:304(304). doi: 10.3389/fphar.2017.00304 28620298 PMC5450331

[77] Sabado RL Pavlick A Gnjatic S Cruz CM Vengco I Hasan F. Resiquimod as an Immunologic Adjuvant for Ny-Eso-1 Protein Vaccination in Patients With High-Risk Melanoma. Cancer Immunol Res (2015) 3(3):278–87. doi: 10.1158/2326-6066.Cir-14-0202 PMC4374362 25633712

[78] Stanley MA. Imiquimod and the Imidazoquinolones: Mechanism of Action and Therapeutic Potential. Clin Exp Dermatol (2002) 27(7):571–7. doi: 10.1046/j.1365-2230.2002.01151.x 12464152

[79] Vasilakos JP Tomai MA. The Use of Toll-Like Receptor 7/8 Agonists as Vaccine Adjuvants. Expert Rev Vaccines (2013) 12(7):809–19. doi: 10.1586/14760584.2013.811208 23885825

[80] Goldinger SM Dummer R Baumgaertner P Mihic-Probst D Schwarz K Hammann-Haenni A. Nano-Particle Vaccination Combined With TLR-7 and -9 Ligands Triggers Memory and Effector CD8^+^ T-Cell Responses in Melanoma Patients. Eur J Immunol (2012) 42(11):3049–61. doi: 10.1002/eji.201142361 PMC3549564 22806397

[81] Speiser DE Schwarz K Baumgaertner P Manolova V Devevre E Sterry W. Memory and Effector CD8 T-Cell Responses After Nanoparticle Vaccination of Melanoma Patients. J Immunother (2010) 33(8):848–58. doi: 10.1097/CJI.0b013e3181f1d614 20842051

[82] Adams S O'Neill DW Nonaka D Hardin E Chiriboga L Siu K. Immunization of Malignant Melanoma Patients With Full-Length NY-ESO-1 Protein Using TLR7 Agonist Imiquimod as Vaccine Adjuvant. J Immunol (2008) 181(1):776–84. doi: 10.4049/jimmunol.181.1.776 PMC2583094 18566444

[83] Ni Q Zhang F Liu Y Wang Z Yu G Liang B. A Bi-Adjuvant Nanovaccine That Potentiates Immunogenicity of Neoantigen for Combination Immunotherapy of Colorectal Cancer. Sci Adv (2020) 6(12):eaaw6071. doi: 10.1126/sciadv.aaw6071 32206706 PMC7080439

[84] Nehete PN Williams LE Chitta S Nehete BP Patel AG Ramani MD. Class C Cpg Oligodeoxynucleotide Immunomodulatory Response in Aged Squirrel Monkey (Saimiri Boliviensis Boliviensis). Front Aging Neurosci (2020) 12:36(36). doi: 10.3389/fnagi.2020.00036 32194391 PMC7063459

[85] Adamus T Kortylewski M. The Revival of CpG Oligonucleotide-Based Cancer Immunotherapies. Contemp Oncology/Współczesna Onkologia (2018) 22(1A):56–60. doi: 10.5114/wo.2018.73887 29628795 PMC5885070

[86] Rothenfusser S Hornung V Ayyoub M Britsch S Towarowski A Krug A. CpG-A and CpG-B Oligonucleotides Differentially Enhance Human Peptide–Specific Primary and Memory CD8^+^ T-Cell Responses *in Vitro* . Blood (2004) 103(6):2162–9. doi: 10.1182/blood-2003-04-1091 14630815

[87] Scheiermann J Klinman DM. Clinical Evaluation of CpG Oligonucleotides as Adjuvants for Vaccines Targeting Infectious Diseases and Cancer. Vaccine (2014) 32(48):6377–89. doi: 10.1016/j.vaccine.2014.06.065 PMC4252359 24975812

[88] Liang Z Cui X Yang L Hu Q Li D Zhang X. Co-Assembled Nanocomplexes of Peptide Neoantigen Adpgk and Toll-Like Receptor 9 Agonist CpG ODN for Efficient Colorectal Cancer Immunotherapy. Int J Pharm (2021) 608:121091. doi: 10.1016/j.ijpharm.2021.121091 34555477

[89] Arbelaez CA Estrada J Gessner MA Glaus C Morales AB Mohn D. A Nanoparticle Vaccine That Targets Neoantigen Peptides to Lymphoid Tissues Elicits Robust Antitumor T Cell Responses. NPJ Vaccines (2020) 5(1):106. doi: 10.1038/s41541-020-00253-9 33298945 PMC7661730

[90] Speiser DE Liénard D Rufer N Rubio-Godoy V Rimoldi D Lejeune F. Rapid and Strong Human CD8^+^ T Cell Responses to Vaccination With Peptide, IFA, and CpG Oligodeoxynucleotide 7909. J Clin Invest (2005) 115(3):739–46. doi: 10.1172/JCI23373 PMC546459 15696196

[91] Pullarkat V Lee PP Scotland R Rubio V Groshen S Gee C. A Phase I Trial of Sd-9427 (Progenipoietin) With a Multipeptide Vaccine for Resected Metastatic Melanoma. Clin Cancer Res (2003) 9(4):1301–12. 12684398

[92] Liénard D Rimoldi D Marchand M Dietrich PY van Baren N Geldhof C. *Ex Vivo* Detectable Activation of Melan-A-Specific T Cells Correlating With Inflammatory Skin Reactions in Melanoma Patients Vaccinated With Peptides in IFA. Cancer Immun (2004) 4:4. doi: 10.1158/1424-9634.DCL-4.4.1 15149168

[93] Baumgaertner P Jandus C Rivals JP Derré L Lövgren T Baitsch L. Vaccination-Induced Functional Competence of Circulating Human Tumor-Specific CD8 T-Cells. Int J Cancer (2012) 130(11):2607–17. doi: 10.1002/ijc.26297 21796616

[94] Tarhini AA Leng S Moschos SJ Yin Y Sander C Lin Y. Safety and Immunogenicity of Vaccination With MART-1 (26-35, 27l), Gp100 (209-217, 210m), and Tyrosinase (368-376, 370d) in Adjuvant With Pf-3512676 and GM-CSF in Metastatic Melanoma. J Immunother (2012) 35(4):359–66. doi: 10.1097/CJI.0b013e31825481fe PMC3483091 22495394

[95] Fourcade J Kudela P Andrade Filho PA Janjic B Land SR Sander C. Immunization With Analog Peptide in Combination With CpG and Montanide Expands Tumor Antigen-Specific CD8^+^ T Cells in Melanoma Patients. J Immunother (2008) 31(8):781–91. doi: 10.1097/CJI.0b013e318183af0b PMC3901357 18779741

[96] Valmori D Souleimanian NE Tosello V Bhardwaj N Adams S O'Neill D. Vaccination With NY-ESO-1 Protein and Cpg in Montanide Induces Integrated Antibody/Th1 Responses and CD8 T Cells Through Cross-Priming. Proc Natl Acad Sci USA (2007) 104(21):8947–52. doi: 10.1073/pnas.0703395104 PMC1885608 17517626

[97] Karbach J Gnjatic S Bender A Neumann A Weidmann E Yuan J. Tumor-Reactive CD8^+^ T-Cell Responses After Vaccination With NY-ESO-1 Peptide, CpG 7909 and Montanide Isa-51: Association With Survival. Int J Cancer (2010) 126(4):909–18. doi: 10.1002/ijc.24850 19728336

[98] Karbach J Neumann A Atmaca A Wahle C Brand K von Boehmer L. Efficient *in Vivo* Priming by Vaccination With Recombinant NY-ESO-1 Protein and CpG in Antigen Naive Prostate Cancer Patients. Clin Cancer Res (2011) 17(4):861–70. doi: 10.1158/1078-0432.Ccr-10-1811 21163871

[99] Baumgaertner P Costa Nunes C Cachot A Maby-El Hajjami H Cagnon L Braun M. Vaccination of Stage III/IV Melanoma Patients With Long NY-ESO-1 Peptide and CpG-B Elicits Robust CD8^+^ and CD4^+^ T-Cell Responses With Multiple Specificities Including a Novel Dr7-Restricted Epitope. Oncoimmunology (2016) 5(10):e1216290. doi: 10.1080/2162402x.2016.1216290 27853637 PMC5087303

[100] Capitini CM Fry TJ Mackall CL. Cytokines as Adjuvants for Vaccine and Cellular Therapies for Cancer. Am J Immunol (2009) 5(3):65–83. doi: 10.3844/ajisp.2009.65.83 20182648 PMC2826803

[101] Malek TR. The Biology of Interleukin-2. Annu Rev Immunol (2008) 26:453–79. doi: 10.1146/annurev.immunol.26.021607.090357 18062768

[102] Hernandez R LaPorte KM Hsiung S Santos Savio A Malek TR. High-Dose IL-2/CD25 Fusion Protein Amplifies Vaccine-Induced CD4^+^ and CD8^+^ Neoantigen-Specific T Cells to Promote Antitumor Immunity. J Immunother Cancer (2021) 9(9):e002865. doi: 10.1136/jitc-2021-002865 34475132 PMC8413969

[103] Fyfe G Fisher RI Rosenberg SA Sznol M Parkinson DR Louie AC. Results of Treatment of 255 Patients With Metastatic Renal Cell Carcinoma Who Received High-Dose Recombinant Interleukin-2 Therapy. J Clin Oncol (1995) 13(3):688–96. doi: 10.1200/JCO.1995.13.3.688 7884429

[104] Atkins MB Lotze MT Dutcher JP Fisher RI Weiss G Margolin K. High-Dose Recombinant Interleukin 2 Therapy for Patients With Metastatic Melanoma: Analysis of 270 Patients Treated Between 1985 and 1993. J Clin Oncol (1999) 17(7):2105–16. doi: 10.1200/JCO.1999.17.7.2105 10561265

[105] Schwartzentruber DJ Lawson DH Richards JM Conry RM Miller DM Treisman J. Gp100 Peptide Vaccine and Interleukin-2 in Patients With Advanced Melanoma. N Engl J Med (2011) 364(22):2119–27. doi: 10.1056/NEJMoa1012863 PMC3517182 21631324

[106] Rosenberg SA Yang JC Schwartzentruber DJ Hwu P Marincola FM Topalian SL. Immunologic and Therapeutic Evaluation of a Synthetic Peptide Vaccine for the Treatment of Patients With Metastatic Melanoma. Nat Med (1998) 4(3):321–7. doi: 10.1038/nm0398-321 PMC2064864 9500606

[107] Smith FO Downey SG Klapper JA Yang JC Sherry RM Royal RE. Treatment of Metastatic Melanoma Using Interleukin-2 Alone or in Conjunction With Vaccines. Clin Cancer Res (2008) 14(17):5610–8. doi: 10.1158/1078-0432.CCR-08-0116 PMC2656367 18765555

[108] Overwijk WW Theoret MR Finkelstein SE Surman DR de Jong LA Vyth-Dreese FA. Tumor Regression and Autoimmunity After Reversal of a Functionally Tolerant State of Self-Reactive CD8^+^ T Cells. J Exp Med (2003) 198(4):569–80. doi: 10.1084/jem.20030590 PMC2194177 12925674

[109] Rosenberg SA Lotze MT Muul LM Chang AE Avis FP Leitman S. A Progress Report on the Treatment of 157 Patients With Advanced Cancer Using Lymphokine-Activated Killer Cells and Interleukin-2 or High-Dose Interleukin-2 Alone. N Engl J Med (1987) 316(15):889–97. doi: 10.1056/nejm198704093161501 3493432

[110] de la Rosa M Rutz S Dorninger H Scheffold A. Interleukin-2 Is Essential for CD4^+^CD25^+^ Regulatory T Cell Function. Eur J Immunol (2004) 34(9):2480–8. doi: 10.1002/eji.200425274 15307180

[111] Tanaka A Sakaguchi S. Targeting Treg Cells in Cancer Immunotherapy. Eur J Immunol (2019) 49(8):1140–6. doi: 10.1002/eji.201847659 31257581

[112] Overwijk WW Tagliaferri MA Zalevsky J. Engineering Il-2 to Give New Life to T Cell Immunotherapy. Annu Rev Med (2021) 72:281–311. doi: 10.1146/annurev-med-073118-011031 33158368

[113] Sharma M Khong H Fa'ak F Bentebibel SE Janssen LME Chesson BC. Bempegaldesleukin Selectively Depletes Intratumoral Tregs and Potentiates T Cell-Mediated Cancer Therapy. Nat Commun (2020) 11(1):661. doi: 10.1038/s41467-020-14471-1 32005826 PMC6994577

[114] Parisi G Saco JD Salazar FB Tsoi J Krystofinski P Puig-Saus C. Persistence of Adoptively Transferred T Cells With a Kinetically Engineered Il-2 Receptor Agonist. Nat Commun (2020) 11(1):660. doi: 10.1038/s41467-019-12901-3 32005809 PMC6994533

[115] Stubsrud E Granum S Zell-Flagstad H Bersaas A Skullerud LM Sekelja M. Abstract 5003: Vaccibody DNA Vaccine Platform VB10.NEO Induces Strong Neo-Antigen Specific CD8^+^ T Cell Responses Critical to Cure Established Tumors in Pre-Clinical Models. Cancer Res (2019) 79(13 Supplement):5003. doi: 10.1158/1538-7445.Am2019-5003

[116] Krieg C Letourneau S Pantaleo G Boyman O. Improved IL-2 Immunotherapy by Selective Stimulation of IL-2 Receptors on Lymphocytes and Endothelial Cells. Proc Natl Acad Sci USA (2010) 107(26):11906–11. doi: 10.1073/pnas.1002569107 PMC2900642 20547866

[117] Jin GH Hirano T Murakami M. Combination Treatment With IL-2 and Anti-IL-2 Mabs Reduces Tumor Metastasis *Via* Nk Cell Activation. Int Immunol (2008) 20(6):783–9. doi: 10.1093/intimm/dxn036 18448458

[118] Levin AM Bates DL Ring AM Krieg C Lin JT Su L. Exploiting a Natural Conformational Switch to Engineer an Interleukin-2 'Superkine'. Nature (2012) 484(7395):529–33. doi: 10.1038/nature10975 PMC3338870 22446627

[119] Tomala J Chmelova H Mrkvan T Rihova B Kovar M. *In Vivo* Expansion of Activated Naive CD8^+^ T Cells and Nk Cells Driven by Complexes of IL-2 and Anti-IL-2 Monoclonal Antibody as Novel Approach of Cancer Immunotherapy. J Immunol (2009) 183(8):4904–12. doi: 10.4049/jimmunol.0900284 19801515

[120] Cho H-I Reyes-Vargas E Delgado JC Celis E. A Potent Vaccination Strategy That Circumvents Lymphodepletion for Effective Antitumor Adoptive T-Cell Therapy. Cancer Res (2012) 72(8):1986–95. doi: 10.1158/0008-5472.Can-11-3246 PMC3328656 22367213

[121] Arenas-Ramirez N Zou C Popp S Zingg D Brannetti B Wirth E. Improved Cancer Immunotherapy by a CD25-Mimobody Conferring Selectivity to Human Interleukin-2. Sci Transl Med (2016) 8(367):367ra166. doi: 10.1126/scitranslmed.aag3187 27903862

[122] Sahin D Arenas-Ramirez N Rath M Karakus U Humbelin M van Gogh M. An IL-2-Grafted Antibody Immunotherapy With Potent Efficacy Against Metastatic Cancer. Nat Commun (2020) 11(1):6440. doi: 10.1038/s41467-020-20220-1 33353953 PMC7755894

[123] Lopes JE Fisher JL Flick HL Wang C Sun L Ernstoff MS. ALKS 4230: A Novel Engineered IL-2 Fusion Protein With an Improved Cellular Selectivity Profile for Cancer Immunotherapy. J Immunother Cancer (2020) 8(1):e000673. doi: 10.1136/jitc-2020-000673 32317293 PMC7204809

[124] Jiang X Wang J Deng X Xiong F Ge J Xiang B. Role of the Tumor Microenvironment in PD-L1/PD-1-Mediated Tumor Immune Escape. Mol Cancer (2019) 18(1):10. doi: 10.1186/s12943-018-0928-4 30646912 PMC6332843

[125] Robert C. A Decade of Immune-Checkpoint Inhibitors in Cancer Therapy. Nat Commun (2020) 11(1):3801. doi: 10.1038/s41467-020-17670-y 32732879 PMC7393098

[126] Liu X Hogg GD DeNardo DG. Rethinking Immune Checkpoint Blockade: ‘Beyond the T Cell’. J Immunother Cancer (2021) 9(1):e001460. doi: 10.1136/jitc-2020-001460 33468555 PMC7817791

[127] Klempner SJ Fabrizio D Bane S Reinhart M Peoples T Ali SM. Tumor Mutational Burden as a Predictive Biomarker for Response to Immune Checkpoint Inhibitors: A Review of Current Evidence. Oncologist (2020) 25(1):e147–e59. doi: 10.1634/theoncologist.2019-0244 PMC6964127 31578273

[128] Yi M Qin S Zhao W Yu S Chu Q Wu K. The Role of Neoantigen in Immune Checkpoint Blockade Therapy. Exp Hematol Oncol (2018) 7:28. doi: 10.1186/s40164-018-0120-y 30473928 PMC6240277

[129] Grosso JF Jure-Kunkel MN. CTLA-4 Blockade in Tumor Models: An Overview of Preclinical and Translational Research. Cancer Immun (2013) 13:5. 23390376 PMC3559193

[130] Davila E Kennedy R Celis E. Generation of Antitumor Immunity by Cytotoxic T Lymphocyte Epitope Peptide Vaccination, CpG-Oligodeoxynucleotide Adjuvant, and CTLA-4 Blockade. Cancer Res (2003) 63(12):3281–8. 12810660

[131] Sorensen MR Holst PJ Steffensen MA Christensen JP Thomsen AR. Adenoviral Vaccination Combined With CD40 Stimulation and CTLA-4 Blockage Can Lead to Complete Tumor Regression in a Murine Melanoma Model. Vaccine (2010) 28(41):6757–64. doi: 10.1016/j.vaccine.2010.07.066 20682365

[132] van Elsas A Hurwitz AA Allison JP. Combination Immunotherapy of B16 Melanoma Using Anti-Cytotoxic T Lymphocyte-Associated Antigen 4 (CTLA-4) and Granulocyte/Macrophage Colony-Stimulating Factor (GM-CSF)-Producing Vaccines Induces Rejection of Subcutaneous and Metastatic Tumors Accompanied by Autoimmune Depigmentation. J Exp Med (1999) 190(3):355–66. doi: 10.1084/jem.190.3.355 PMC2195583 10430624

[133] Curran MA Allison JP. Tumor Vaccines Expressing Flt3 Ligand Synergize With CTLA-4 Blockade to Reject Preimplanted Tumors. Cancer Res (2009) 69(19):7747–55. doi: 10.1158/0008-5472.Can-08-3289 PMC2756314 19738077

[134] Curran MA Montalvo W Yagita H Allison JP. PD-1 and CTLA-4 Combination Blockade Expands Infiltrating T Cells and Reduces Regulatory T and Myeloid Cells Within B16 Melanoma Tumors. Proc Natl Acad Sci USA (2010) 107(9):4275–80. doi: 10.1073/pnas.0915174107 PMC2840093 20160101

[135] Yuan J Ginsberg B Page D Li Y Rasalan T Gallardo HF. CTLA-4 Blockade Increases Antigen-Specific CD8^+^ T Cells in Prevaccinated Patients With Melanoma: Three Cases. Cancer Immunol Immunother (2011) 60(8):1137–46. doi: 10.1007/s00262-011-1011-9 PMC3654853 21465316

[136] Hailemichael Y Dai Z Jaffarzad N Ye Y Medina MA Huang X-F. Persistent Antigen at Vaccination Sites Induces Tumor-Specific CD8^+^ T Cell Sequestration, Dysfunction and Deletion. Nat Med (2013) 19(4):465–72. doi: 10.1038/nm.3105 PMC3618499 23455713

[137] Hailemichael Y Fu T Khong H Dai Z Sharma P Overwijk WW. Reversing Gp100/Ifa-Induced Impairment of Anti-CTLA-4 Checkpoint Blockade Therapy. J Immunother Cancer (2014) 2(Suppl 3):P14. doi: 10.1186/2051-1426-2-s3-p14

[138] Dong Y Sun Q Zhang X. PD-1 and Its Ligands Are Important Immune Checkpoints in Cancer. Oncotarget (2017) 8(2):2171–86. doi: 10.18632/oncotarget.13895 PMC5356790 27974689

[139] Henick BS Herbst RS Goldberg SB. The PD-1 Pathway as a Therapeutic Target to Overcome Immune Escape Mechanisms in Cancer. Expert Opin Ther Targets (2014) 18(12):1407–20. doi: 10.1517/14728222.2014.955794 25331677

[140] Pedoeem A Azoulay-Alfaguter I Strazza M Silverman GJ Mor A. Programmed Death-1 Pathway in Cancer and Autoimmunity. Clin Immunol (2014) 153(1):145–52. doi: 10.1016/j.clim.2014.04.010 24780173

[141] Swart M Verbrugge I Beltman JB. Combination Approaches With Immune-Checkpoint Blockade in Cancer Therapy. Front Oncol (2016) 6:233. doi: 10.3389/fonc.2016.00233 27847783 PMC5088186

[142] Robert C Schachter J Long GV Arance A Grob JJ Mortier L. Pembrolizumab Versus Ipilimumab in Advanced Melanoma. N Engl J Med (2015) 372(26):2521–32. doi: 10.1056/NEJMoa1503093 25891173

[143] Chung V Kos FJ Hardwick N Yuan Y Chao J Li D. Evaluation of Safety and Efficacy of P53MVA Vaccine Combined With Pembrolizumab in Patients With Advanced Solid Cancers. Clin Transl Oncol (2019) 21(3):363–72. doi: 10.1007/s12094-018-1932-2 PMC8802616 30094792

[144] Kunimura N Kitagawa K Sako R Narikiyo K Tominaga S Bautista DS. Combination of Rad-P53 *in Situ* Gene Therapy and Anti-PD-1 Antibody Immunotherapy Induced Anti-Tumor Activity in Mouse Syngeneic Urogenital Cancer Models. Sci Rep (2020) 10(1):17464. doi: 10.1038/s41598-020-74660-2 33060772 PMC7562933

[145] Gibney GT Kudchadkar RR DeConti RC Thebeau MS Czupryn MP Tetteh L. Safety, Correlative Markers, and Clinical Results of Adjuvant Nivolumab in Combination With Vaccine in Resected High-Risk Metastatic Melanoma. Clin Cancer Res (2015) 21(4):712–20. doi: 10.1158/1078-0432.CCR-14-2468 PMC4620684 25524312

[146] Kos S Lopes A Preat V Cemazar M Lampreht Tratar U Ucakar B. Intradermal DNA Vaccination Combined With Dual CTLA-4 and PD-1 Blockade Provides Robust Tumor Immunity in Murine Melanoma. PloS One (2019) 14(5):e0217762. doi: 10.1371/journal.pone.0217762 31150505 PMC6544376

